# Dorsal Root Ganglion Maintains Stemness of Bone Marrow Mesenchymal Stem Cells by Enhancing Autophagy through the AMPK/mTOR Pathway in a Coculture System

**DOI:** 10.1155/2018/8478953

**Published:** 2018-09-30

**Authors:** Shuaishuai Zhang, Junqin Li, Huijie Jiang, Yi Gao, Pengzhen Cheng, Tianqing Cao, Donglin Li, Jimeng Wang, Yue Song, Bin Liu, Hao Wu, Chunmei Wang, Liu Yang, Guoxian Pei

**Affiliations:** ^1^Department of Orthopaedics, Xijing Hospital, Fourth Military Medical University, Xi'an 710032, China; ^2^Department of Orthopaedics, The 463rd Hospital of PLA, Shenyang 110042, China; ^3^Department of Orthopaedics, The 251st Hospital of PLA, Zhangjiakou 075000, China

## Abstract

Our previous studies found that sensory nerve tracts implanted in tissue-engineered bone (TEB) could result in better osteogenesis. To explore the mechanism of the sensory nerve promoting osteogenesis in TEB in vitro, a transwell coculture experiment was designed between dorsal root ganglion (DRG) cells and bone marrow mesenchymal stem cells (BMSCs). BMSC proliferation was determined by CCK8 assay, and osteo-, chondro-, and adipogenic differentiation were assessed by alizarin red, alcian blue, and oil red staining. We found that the proliferation and multipotent differentiation of BMSCs were all enhanced in the coculture group compared to the BMSCs group. Crystal violet staining showed that the clone-forming ability of BMSCs in the coculture group was also enhanced and mRNA levels of Sox2, Nanog, and Oct4 were significantly upregulated in the coculture group. Moreover, the autophagy level of BMSCs, regulating their stemness, was promoted in the coculture group, mediated by the AMPK/mTOR pathway. In addition, AMPK inhibitor compound C could significantly downregulate the protein expression of LC3 and the mRNA level of stemness genes in the coculture group. Finally, we found that the NK1 receptor antagonist, aprepitant, could partly block this effect, which indicated that substance P played an important role in the effect. Together, we conclude that DRG could maintain the stemness of BMSCs by enhancing autophagy through the AMPK/mTOR pathway in a transwell coculture system, which may help explain the better osteogenesis after implantation of the sensory nerve into TEB.

## 1. Introduction

Bone tissue engineering has provided a promising resolution for the treatment of large bone defect, although there are still many problems to be solved [1–3], such as low survival rate and poor osteogenic differentiation of BMSCs. Previously, we found that the sensory nerve tract preimplanted in the tissue-engineered bone (TEB) could significantly improve osteogenesis of the TEB [4, 5], but the underlying mechanism was still largely unknown.

The sensory nerve has been reported to play critical roles in bone metabolism and regeneration in vivo [6–9]. Some studies found that sensory nerve innervation contributed to the maintenance of trabecular bone mass and its mechanical properties by inhibiting bone resorption [8]. Besides, sensory nerves had efferent functions in the tissues they innervated, mediated by transmitters released from the peripheral nerve terminals, which helped to maintain trabecular bone integrity [10]. Recently, sensory neuron-derived Sema3A was found to be responsible for the bone mass loss in Sema3a^−/−^ mice, and Sema3A regulated bone remodelling indirectly by modulating sensory nerve innervation, not directly by acting on osteoblasts [7].

Studies in vitro indicated that neuropeptides, such as substance P and CGRP [11], could influence preosteoblast cells. For example, SP significantly increased proliferation of BMSCs in vitro in a dose-dependent manner [12]. And SP could induce osteoblastic differentiation of BMSCs via the Wnt/*β*-catenin pathway and promote the angiogenic ability of BMSCs [13]. In addition, CGRP was reported to exert its anabolic action on human osteoblasts by stimulating canonical Wnt signaling and inhibiting human osteoblast apoptosis [14].

Taken together, these findings in vivo and in vitro suggested that the sensory nerve played a vital role in bone metabolism and regeneration. However, the mechanism at the cellular level is rarely studied, and one kind of neuropeptide may not reflect the overall effect of the sensory nerve on bone cells. Therefore, the overall regulation of the sensory nerve on bone cells and its underlying molecular mechanisms need further studies.

In this study, we used a transwell coculture system to investigate the effect of DRG cells on BMSCs. Our results indicated that DRG could help maintain the stemness of BMSCs through improving the basal autophagy level by activating AMPK/mTOR signaling in this coculture system.

## 2. Materials and Methods

### 2.1. Isolation and Characterization of GFP Rat BMSCs

BMSCs from 2-week-old GFP Sprague–Dawley (SD) rats were harvested using a well-characterized protocol with slight modification [15]. Briefly, primary cultures of BMSCs were obtained from a 2-week-old GFP rat under sterile conditions. The rats were sacrificed with an overdose of 2% pentobarbital sodium (*w*/*v*). After immersing in 75% ethanol (*v*/*v*) for 5 min, both the femurs and tibias were isolated and excised with the attached soft tissues, and then epiphyses were cut off with aseptic scissors. Then, the marrow was flushed from the diaphysis with a syringe and collected in a primary culture medium (*α*-MEM containing 10% FBS and 1% antibiotics (penicillin and streptomycin)). The collected medium containing marrow cells was cultured in 6-well culture plates with 2 ml *α*-MEM containing 10% FBS each well. After 24 hours, nonadherent cells were carefully removed. The cell growth medium was changed every 2 days, and cells were subcultured until reaching 80% confluence. Passage 3 BMSCs were seeded in a 6-well culture plate at a concentration of 2 × 10^3^ cells/ml for coculture with DRG. BMSCs from passage 3 were characterized with flow cytometry and pluripotent differentiation.

### 2.2. Flow Cytometry

BMSCs from passage 3 were trypsinized, centrifuged, and suspended in cold PBS, and the cell number was calculated. Then, BMSCs were blocked with 2% BSA for 30 min at room temperature. Meanwhile, the antibodies were diluted to the recommended concentration with PBS. After that, cell density was adjusted to 1 × 10^6^ each tube. Then, cells were incubated for 40 min at 4°C with a PerCP anti-rat CD90 antibody (202512, BioLegend), PE anti-rat CD11b/c antibody (201807, BioLegend), PE anti-rat CD34 antibody (ab187284, Abcam), and PE-Cy7 anti-rat CD45 antibody (202214, BioLegend). The incubated cells were analyzed by flow cytometry (cytomics FC 500, Beckman Coulter) using isotype-identical antibodies as controls.

### 2.3. Preparation of DRG and Coculture between BMSCs and DRG Cells

One-day-old postnatal SD rats were sacrificed, and isolation of DRG was based on the previous study [16]. The vertebral column was exposed and opened from the thoracic to the lumbar region. Then, DRG was dissected from the lumbar spinal cord and washed with ice-cold PBS. The connective tissue was carefully removed. DRG was pooled in *α*-MEM medium on ice until further procedure. The collected DRG was cut mechanically with scissors for 2 minutes and then incubated for 30 minutes at 37°C with 0.1% (*v*/*v*) trypsin. Then, cells were filtered through a 100 *μ*m cell strainer and centrifuged at 180*g* for 5 minutes. The harvested DRG cells were planted in the transwell cell insert (Lot 3450, Corning, USA), which had already been put into the 6-well culture plate, at a concentration of 1 × 10^4^ cells per well. Then, 1 ml of the cell growth medium was added into the transwell insert, which meant that there was 3 ml of the cell growth medium in all 6 wells of the plate. BMSCs cultured without DRG cells were used as the control group. They were cultured at 37°C in 5% CO_2_ atmosphere. The use of transwell cell inserts allowed DRG cells and BMSCs to share the same cell growth medium but had no direct contact.

### 2.4. BMSC Osteogenic Differentiation and Alizarin Red Staining

The osteogenic differentiation medium (RASMX-90021) was purchased from Cyagen Biosciences Inc., and the procedure was performed according to the products' user manual. Briefly, after coculture for 8 days, BMSCs were detached and seeded on a new 12-well culture plate at the density of 2 × 10^4^ cells/well. The osteogenic medium consisted of DMEM with 10% FBS, 50 mg/ml ascorbic acid-2phosphate, 100 nM dexamethasone, and 10 mM *β*-glycerol phosphate in the presence of 100 U/ml penicillin and 100 mg/ml streptomycin. When cells were approximately 60–70% confluent, the growth mediums were carefully aspirated off from each well and 2 ml of the osteogenic differentiation medium was added. The osteogenic induction mediums were changed every 3 days. After 14 days, cells could be fixed with 4% paraformaldehyde and stained with Alizarin red S (Cyagen Biosciences Inc.) for 3 to 5 min, and then cells were observed under the microscope.

### 2.5. BMSC Chondrogenic Differentiation and Alcian Blue Staining

For chondrogenic differentiation, 0.5 ml BMSCs containing 2.5 × 10^5^ cells is needed to form one chondrogenic pellet in 15 ml polypropylene culture tubes with the differentiation induction medium (RASMX-90041, Cyagen Biosciences, USA). The caps of the tubes were loosened in order to allow gas exchange, and cells were incubated at 37°C in a humidified atmosphere of 5% CO_2_. The pellets were not disturbed for 24 hours. The chondrogenic induction mediums were changed every 2–3 days in each tube (to avoid aspirating the pellets when aspirating the medium, attach a sterile 1–200 *μ*l pipette tip to the end of the aspirating pipette). 0.5 ml of the freshly prepared complete chondrogenic medium was added to each tube. To ensure that the pellet was free floating, the bottom of the tube was flicked several times. Chondrogenic pellets were harvested after 20 days of culture. Pellets were formalin-fixed, and frozen sections of 8 *μ*m thickness were obtained for alcian blue staining analysis.

### 2.6. Adipogenic Differentiation and Oil Red Staining

For adipogenic differentiation, the differentiation induction medium (RASMD-90031, 1 *μ*M DEX, 1 *μ*g/ml insulin, and 0.5 mM 3-isobutyl-1-methylxanthine) and differentiation basal medium (glutamine, insulin) were prepared according to the user's manual. On the 8th day of experiment, BMSCs were reseeded at 2 × 10^4^ cells/cm^2^ in a 6-well tissue culture plate with a medium volume of 2 ml per well. Upon reaching 100% confluent or postconfluent, 2 ml of the induction medium was added per well. Three days later, the medium was changed to a maintenance medium. 24 hours later, the medium was changed back to the induction medium. After repeating the cycle for 4 times, the maintenance medium was continuously used for 4 to 7 days. After the cells had differentiated, cells were rinsed and fixed with 4% paraformaldehyde. 1 ml of oil red O (Cyagen Biosciences Inc.) working solution was added (diluted to 3 : 2 with distilled water and filtered with a filter paper) for 30 minutes to each well. Then, cells were observed under a microscope.

### 2.7. Cell Proliferation Assay

Cell proliferation assay was performed as previously described [17]. Briefly, cells were seeded on 6-well plates (10^4^ cells/well) and cultured with or without DRG for indicated time lengths. At days 1, 2, 4, 6, 8, and 10, the Cell Counting Kit-8 (Dojindo) was applied and incubated for 2 hours, and then absorbance of formazan dye produced by living cells at 450 nm was measured with a microplate reader (Synergy H1, BioTek, USA). All experiments were performed three separate times.

### 2.8. Colony-Forming Unit (CFU) Assay

CFU assay was conducted to measure the self-renewal ability of BMSCs with or without DRG cells. Briefly, BMSCs, after coculture with DRG for 8 days, were seeded at the number of 50 cells in a 60 mm plate and cultured for 10 days, then stained with crystal violet staining solution. Colonies containing more than 50 cells were counted under a microscope. Monocultured BMSCs were recognized as the control in all the experiments. All experiments were performed three separate times.

### 2.9. Real-Time Quantitative PCR

All procedures were performed according to products' instruction and referring to a well-documented method in a previous study [15]. Briefly, total RNA from BMSCs was extracted using OMEGA Total RNA Kit I (lot R6834-01, OMEGA bio-tek, USA) according to the manufacturer's protocol. Concentration and purity of the RNA were determined by measuring the absorbance in TE buffer (10 mM Tris-HCl, pH 8.0, and 1 mM EDTA) at 260 and 280 nm. Then, cDNA was synthesized from the total RNA using a Takara PrimeScript™ RT Master Mix (Perfect Real Time) kit (Lot RR036, Takara, Japan) following the supplier's instructions. The levels of mRNA of Sox2, Nanog, and Oct4 in BMSCs were determined by quantitative real-time RT-PCR using the Takara SYBR Green I kit according to the user manual (Bio-Rad, CFX96, Real-Time System, USA). The sequences of the primers are as follows: for Sox2, forward primer 5′-GTCAGCGCCCTGCAGTACAA-3′ and reverse primer 5′-GCGAGTAGGACATGCTGTAGGTG-3′; for Oct4, forward primer 5′-GACAACCATCTGCCGCTTC-3′ and reverse primer 5′-TCCTCCACCCACTTCTCCA-3′; for Nanog, forward primer 5′-TGGACACTGGCTGAATCCTTC-3′ and reverse primer 5′-CGCTGATTAGGCTCCAACCAT-3′; and for GADPH (as the internal control), forward primer 5′-ACAGGGCTATCAGGGAGCA-3′ and reverse primer 5′-GGAGCGAGATCCCTCCAAAAT-3′.

### 2.10. Western Blotting Analysis

Western blotting analysis was conducted as previously described [18]. Briefly, BMSCs in the 6-well plate were washed in cold-buffered PBS and lysed in RIPA buffer with 1 mm PMSF on ice. Cell lysates were centrifuged (12000 rpm, 10 min) at 4°C, and the protein supernatant was transferred into new tubes. The concentration of the protein samples was determined with the BCA Protein Assay Kit (PC0020, Solarbio, Beijing, China). A 20 *μ*g sample of the total protein was resolved using 12% SDS-PAGE and transferred onto PVDF membranes. The membranes were blocked in Tris-buffered saline containing Tween 20 with 5% BSA at room temperature for 2 h. Primary antibodies (LC3A/B, 50 *μ*g/50 *μ*l, AF5402, anti-LC3A/B antibody, 1 : 1000, Affinity, USA; AMPK, ab32047, 1 : 2500, Abcam, USA; p-AMPK, ab133448, 1 : 5000, Abcam, USA; P-AKT, ab81283, 1 : 7000, Abcam, USA; AKT, ab8805, 1 : 500, Abcam, USA; mTOR, ab2732, 1 : 2000, Abcam, USA; P-mTOR, ab137133, 1 : 5000, Abcam, USA; and *β*-actin, 66009-1-lg, 1 : 20000, Proteintech Group, USA) were incubated overnight with the membranes at 4°C. Membranes were incubated with horseradish peroxidase-conjugated anti-rabbit secondary antibodies (Goat Anti-Rabbit IgG (HRP), Abcam, ab6721, USA), and proteins were detected by enhanced chemiluminescence (Beyotime, Shanghai, China) using Amersham Imager 600 (General Electric Company, USA). *β*-Actin was used as the internal control to normalize the loading materials.

### 2.11. Immunofluorescence

Cells were immobilized with 4% paraformaldehyde and permeabilized with 1% triton X-100 for 10 min, followed by blocking with 2% bovine serum albumin for 30 minutes. Then, the primary antibody, rabbit polyclonal antibody (LC3A/B, 50 *μ*g/50 *μ*l, AF5402, anti-LC3A/B antibody, 1 : 100, Affinity, USA), was used for incubation overnight at 4°C. Then, cells were incubated with a secondary antibody Alexa Fluor 647 donkey anti-rabbit (ab150075, 1 : 200, Abcam, USA) away from light for 1 hour. After that, cells were mounted with DAPI (1 : 1000, 32670-5MG-F, Sigma, USA) for 5 minutes. Immunofluorescent images were captured and analyzed with a confocal microscope (Olympus, FV10-ASW3.1, JPN).

### 2.12. Drug Treatment

The AMPK inhibitor, compound C, was purchased from Millipore (Merck, Billerica, MA, USA), and the dose of compound C was 20 *μ*Μ [19, 20]. Compound C was added into the system for 24 hours after coculture for 8 days. The NK1 receptor antagonist, aprepitant, was purchased from Sigma-Aldrich (SML2215-5MG, Shanghai, China). The dose of aprepitant was 50 *μ*Μ [21, 22]. After coculture for 8 days, DRG and BMSCs were treated with aprepitant for 48 hours. Then, BMSCs were collected for the following analysis.

### 2.13. Statistical Analysis

All data were expressed as means ± standard deviation and analyzed by SPSS software (version 13.0). The difference between groups was compared by Student's *t*-test, and *P* value less than 0.05 was considered to be statistically significant.

## 3. Results

### 3.1. Characterization of GFP Rat BMSCs

**Figure 1:**
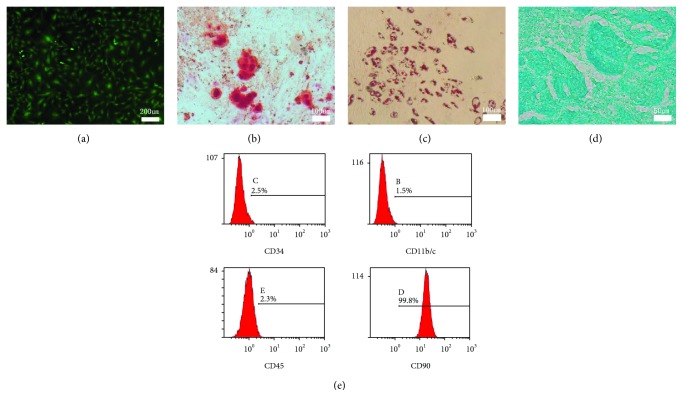
Characterization of GFP BMSCs. (a) Morphology of P3 GFP BMSCs. (b) Alizarin red staining of BMSCs after osteogenic induction for 14 days. (c) Oil red staining of BMSCs after adipogenic induction for 10 days. (d) Alcian blue staining of BMSCs after chondrogenic induction for 20 days. (e) Representative results of flow cytometry analysis of P3 BMSCs indicating abundant expression of CD90 and the absence of CD 34, CD11b/c, and CD45.

BMSCs appeared to have fusiform morphology with green fluorescence, the classical form of them (Figure 1(a)). The multipotential differentiation of BMSCs was verified by alizarin red staining (Figure 1(b)), oil red O staining (Figure 1(c)), and alcian blue staining (Figure 1(d)), which showed that BMSCs used in this study could differentiate into osteoblasts, adipocytes, and chondrocytes under induction condition. The flow cytometry analysis suggested that P3 cells used in this experiment had abundant expression of CD90 (99.8%) and the absence of CD34 (2.5%), CD11b/c (1.5%), and CD45 (2.3%) (Figure 1(e)). Together, BMSCs in this study had multiple potential for differentiation, and the purity of BMSCs was very high, so P3 BMSCs could be used for the following experiments.

### 3.2. Coculturing with DRG Promoted Proliferation and Enhanced Multipotential Differentiation of BMSCs

**Figure 2:**
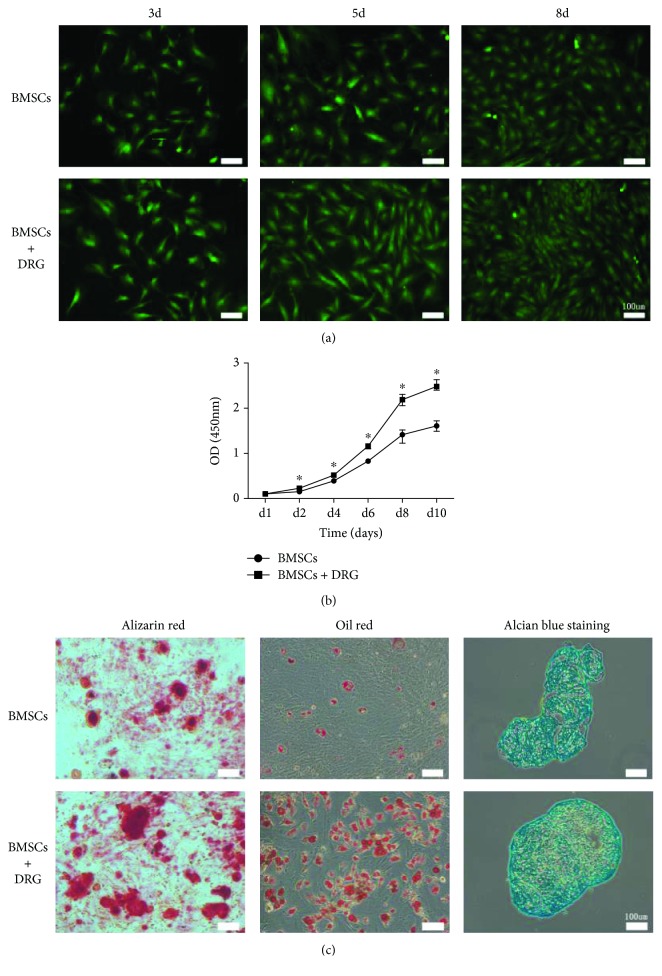
Coculturing with DRG promoted proliferation and enhanced multipotential differentiation of BMSCs. (a) Morphology and density of GFP BMSCs at days 3, 5, and 8. (b) Proliferation curves of BMSCs after coculture with DRG by the CCK8 assay. (c) Osteogenic, adipogenic, and chondrogenic differentiation of BMSCs were significantly promoted after coculture with DRG for 8 days.

Here, we analyzed the effect of coculturing with DRG on BMSC proliferation and multipotential differentiation. We found that coculturing with DRG significantly promoted BMSC proliferation and elevated their osteogenic, adipogenic, and chondrogenic differentiations. Rat GFP-BMSCs in the coculture group showed similar fibroblastic morphology to those from the control group (Figure 2(a)). However, the density of BMSCs in the coculture group was larger than that in the control group at distinct time points (day 3, day 5, and day 8) (Figure 2(a)). To assess the proliferation of the two groups, CCK8 analysis was conducted until day 10, and the BMSC proliferation curve showed that BMSC coculturing with DRG proliferated more significantly at each time point, especially at day 6 and day 8, compared with the control BMSC group (Figure 2(b)). So we chose BMSCs at coculture day 8 to analyze the effect of DRG on multiple differentiation of BMSCs. The results of alizarin red, oil red O, and alcian blue stainings showed that osteogenic, adipogenic, and chondrogenic differentiations of BMSCs were all enhanced in the DRG + BMSC group, compared with the BMSC group (Figure 2(c)). These findings suggested that coculture with DRG could not only enhance proliferation but also promote multiple differentiation of BMSCs, which hinted that coculture with DRG may have an effect on stemness of BMSCs.

### 3.3. Coculturing with DRG Promoted the Self-Renewal Ability and Stem Cell-Related Gene Expression of BMSCs

**Figure 3:**
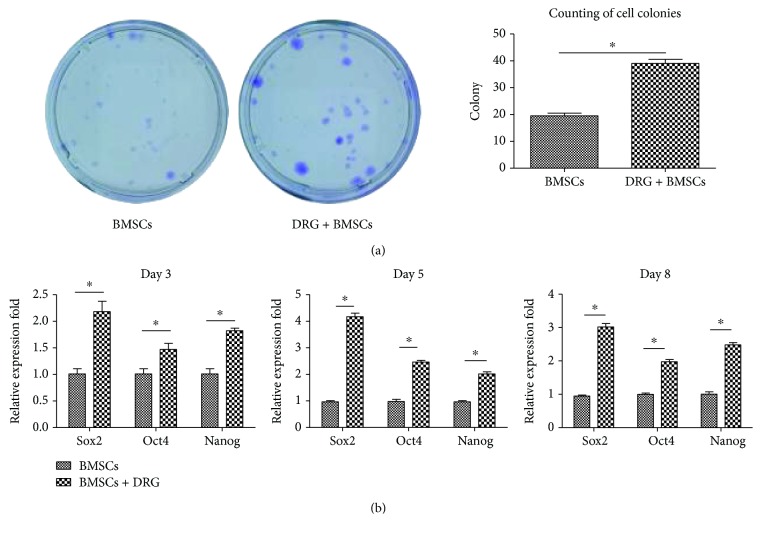
Coculturing with DRG promoted the self-renewal ability and stem cell-related gene expression of BMSCs. (a) CFU assay of the BMSCs and BMSCs + DRG groups. Purple dots refer to cell colonies. (b) Stem cell-related gene expression after coculture for 3, 5, and 8 days. ∗ denotes that differences are statistically significant between BMSCs + DRG and BMSCs groups.

To investigate the effect of coculturing with DRG on stemness of BMSCs, CFU assay, which detected the self-renewal ability of BMSCs, was conducted. BMSCs at day 8 were used and cultured for 10 days. Compared with the BMSC group, more colonies were formed and the colony area was much larger in the DRG + BMSC group (*P* < 0.05, Figure 3(a)). To verify the effect of DRG on BMSC stemness, we detected the expression of stem cell-related genes, Nanog, Oct4, and Sox2, and all of them were significantly upregulated after coculture for 3, 5, and 8 days, especially at days 5 and 8 (*P* < 0.05, Figure 3(b)), which was in line with the result of BMSC proliferation. These findings indicated that coculture with DRG could maintain the stemness of BMSCs.

### 3.4. Coculturing with DRG Enhanced BMSC Autophagy

**Figure 4:**
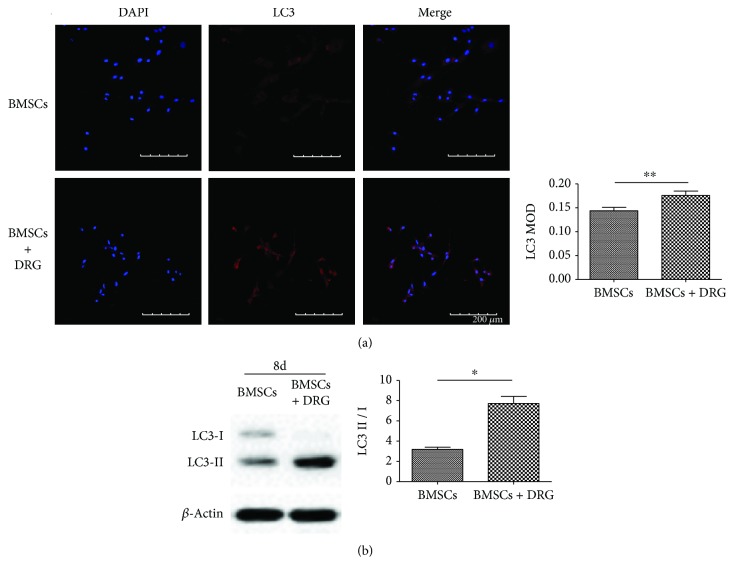
Coculturing with DRG enhanced BMSCs autophagy. (a) LC3 immunofluorescence images after coculture for 8 days. (b) Western blot analysis of LC3II and LC3I and the LC3II/LC3I ratio analysis after coculture for 8 days. MOD: mean optical density. ∗ denotes that differences are statistically significant between BMSCs + DRG and BMSCs groups.

As was indicated by mounting evidence, autophagy plays a critical role in the stemness maintenance of stem cells. To verify whether autophagy was involved in the process of stemness maintenance of BMSCs in the coculture group, we detected the protein expression of autophagic markers LC3II and LC3I by Western blot and immunofluorescence at day 8. BMSCs in the coculture group displayed stronger LC3 fluorescence intensity than BMSCs in the control group (Figure 4(a)). The conversion of soluble LC3I to lipid-bound LC3II is an indicator of autophagosome formation, so the LC3II/LC3I ratio means the level of autophagy activation. Western blot results showed that there was much more LC3II protein expression in the coculture group and the LC3II/LC3I ratio was predominantly larger in the coculture group than in the control group (Figure 4(b)). These results suggested that autophagy was activated in BMSCs during the process of coculture with DRG.

### 3.5. AMPK/mTOR Signaling Was Activated in the Coculture Group

**Figure 5:**
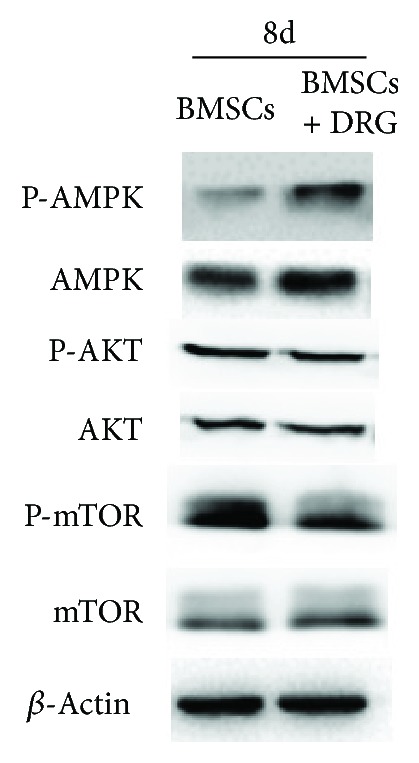
AMPK signaling pathway, not the AKT signaling pathway, was activated in BMSCs after coculture for 8 days.

In order to identify the mechanism of autophagy activation of BMSCs in the coculture group, we next investigated two classical signaling pathways involved in autophagy activation, including AMPK/mTOR and AKT/mTOR signaling pathways, by Western blot after coculture for 8 days. The activation of mTOR could inhibit the autophagy, and phosphorylation of AMPK inhibited the activation of mTOR, so the activation of AMPK would promote the level of autophagy. The results showed that the protein expression of p-AMPK in the coculture group was significantly upregulated compared to that in the control group, while the expression of P-AKT remained unchanged between two groups (Figure 5). These results provided evidence that autophagy activation of BMSCs in the coculture group may be mediated through AMPK/mTOR signaling but not through AKT/mTOR signaling.

### 3.6. Compound C Treatment Downregulated Autophagy and Stem Cell-Related Genes in the Coculture Group

**Figure 6:**
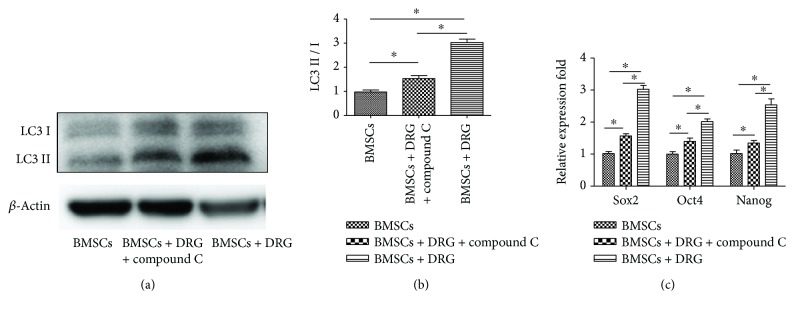
Compound C treatment downregulated autophagy and stem cell-related genes in the coculture group. (a) Western blot analysis of LC3II and LC3I after treatment with compound C. (b) Analysis of LC3II/LC3I among the three groups. (c) Stem cell-related gene expression after treatment with compound C. ∗ denotes that differences are statistically significant between two groups. Compound C alone did not change the autophagy level or stemness genes of BMSCs (Fig S1).

To further confirm whether AMPK/mTOR signaling mediated the autophagy and maintenance of BMSC stemness, we treated BMSCs with compound C, an AMPK-specific inhibitor, in the coculture group. After treatment with compound C for 8 days, the protein expression of LC3II and LC3I was significantly decreased compared with the coculture group without compound C (*P* < 0.05, Figures 6(a) and 6(b)). This indicated that AMPK/mTOR signaling mediated the autophagy of BMSCs in the coculture system. Moreover, the mRNA levels of stemness genes, Sox2, Oct4, and Nanog, were predominantly downregulated, yet still higher than those of the control group (*P* < 0.05, Figure 6(c)). These data demonstrated that autophagy mediated by AMPK/mTOR signaling took an active part in BMSC stemness maintenance in the coculture group, although not the whole part, as the expression of stem cell-related genes in the coculture + compound C group was still higher than that in the control group.

**Figure 7:**
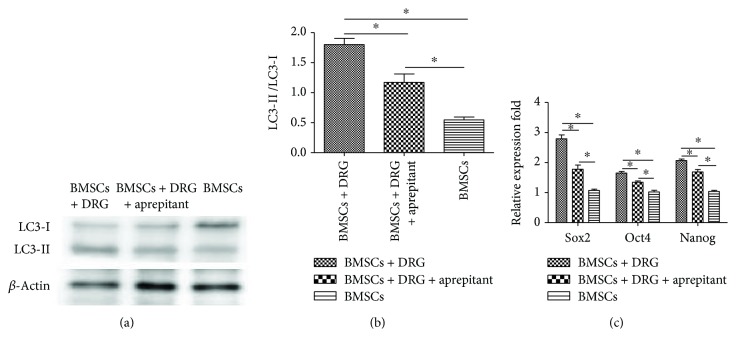
Effect of aprepitant on autophagy and stemness genes in the coculture system. (a) Western blot analysis of LC3I and LC3II after treatment with aprepitant. (b) Analysis of LC3II/LC3I among the three groups. (c) Stemness gene expression after treatment with aprepitant. ∗ denotes that differences are statistically significant between two groups.

To identify the factors involved in the effects of DRG-derived cells on BMSCs, DRG and BMSCs were cocultured for 8 days and then were treated with aprepitant, an NK1 receptor antagonist, for 48 h at the concentration of 50 *μ*Μ. Then, BMSCs were collected, and autophagy-related proteins LC3I and LC3II and stemness gene expressions in the three groups, BMSCs + DRG, BMSCs + DRG + aprepitant, and BMSCs, were detected. We found that the expression of autophagy-related proteins and the ratio of LC3II/I decreased after adding aprepitant into the coculture system (Figures 7(a) and 7(b)), but the autophagy protein expression and the ratio of LC3II/I were still higher than those of the control group (Figures 7(a) and 7(b)). Similar to the autophagy level, the stemness genes were also downregulated after treatment with aprepitant compared with the coculture group but were still higher than those of the control group (Figure 7(c)). These results showed that aprepitant could partly block the effect of DRG-derived cells on BMSCs, which indicated that substance P might play an important role in the effect of DRG-derived cells on BMSCs in the coculture system.

## 4. Discussion

A number of studies, both in vivo and in vitro, have explored the diverse roles of the sensory nerve in bone physiology [6–10]. However, the direct effect of the sensory nerve on bone cells at the cellular level and its underlying molecular mechanisms were still unclear. In order to investigate the overall effect of the sensory nerve on cell biological behavior of BMSCs, we designed a transwell coculture system between DRG and BMSCs, considering that there was no direct contact between DRG and BMSCs in the bone in vivo. We found that DRG could promote the proliferation and self-renewal ability of BMSCs. What is more, multipotential differentiation of BMSCs was more significant in the coculture group. Since the ability of self-renewal and multiple differentiation potential were two major characteristics of stem cells [23], we assumed that coculture with DRG might help keep the stemness of BMSCs. Next, we found that stem cell-related genes, Sox2, Nanog, and Oct4, were upregulated in the coculture group, which further confirmed the role of DRG in stemness maintenance of BMSCs in this coculture system.

As was reported, the stemness maintenance of stem cells had a close relationship with autophagy [24–27]. Autophagy is a process of self-degradation of cellular components in which double-membrane autophagosomes sequester organelles or portions of cytosol and fuse with lysosomes or vacuoles for breakdown by resident hydrolases [28]. The mechanism of autophagy maintaining stemness was studied in many researches. They concluded that autophagy helped maintain stemness through suppressing stem cell metabolism by clearing active, healthy mitochondria [25], clearing away toxic cellular waste [26], and preventing reactive oxygen species (ROS) accumulation [27]. In the process of autophagy, LC3 was reported to be a well-known marker of autophagosomes, and the ratio of LC3II/LC3I reflected the level of autophagy [26]. In our study, to confirm whether autophagy played a role in the maintenance of stemness of BMSCs in the coculture system, the expression of LC3 was detected and the ratio of LC3II/LC3I was analyzed. Our results proved that the basal autophagy level in the coculture group was higher than that in the control group, but we still could not conclude that the higher autophagy level resulted in the maintenance of BMSCs in this study.

Mounts of evidences have revealed the signaling regulation of autophagy [29, 30], among which AMPK/mTOR and AKT/mTOR were two most studied pathways [31–34]. So we examined the protein expression of AMPK, AKT, and mTOR at day 8 during coculture, and we found that, compared to the control group, p-AMPK was upregulated and mTOR was downregulated, while AKT was unchanged in the coculture group. Therefore, AMPK/mTOR signaling mediated the enhancing autophagy. What is more, compound C, an AMPK inhibitor [35, 36], downregulated the autophagy and stem cell-related genes in the coculture group, which demonstrated that autophagy maintained the stemness of BMSCs in the coculture group mediated by the AMPK/mTOR pathway. In addition, the stemness genes in the compound C group, although lower than those in the DRG coculture group, were still higher than those in the BMSC monoculture group, which might suggest that there existed other mechanisms of stemness maintenance in the coculture group except the enhanced autophagy.

In order to further determine the factors by which DRG acts on BMSCs, two major sensory neuropeptides, SP and CGRP, were taken into consideration. However, according to some researches, CGRP receptors exist not only in BMSCs but also in DRG and Schwann cells, and CGRP can play an important role in the function of Schwann cells and DRG [37–39]. Therefore, in the coculture system, if the CGRP receptor inhibitor is added, it would not only block the CGRP receptors of BMSCs but also block the CGRP receptors of Schwann cells and DRG neurons, which would totally change the original system and make the results hard to analyze. Since we did not find that SP had any effect on DRG and Schwann cells, we used the NK1 receptor antagonist, aprepitant [21, 22], to explore the role of SP in this coculture system.

In several studies, the coculture system had been used to investigate the communication between neurons and other cells [16, 40–43]. It was reported that DRG could promote the proliferation of osteoblasts differentiated from BMSCs and the osteogenic gene expression under direct contact condition [40]. And the sensory neuron could regulate MC3T3-E1 cells through exocytosis of glutamate and substance P, and it was confirmed that the peptidergic neurons were involved in this process [43]. More recently, Silva et al. [41] designed a microfluidic device where only the neurites from DRG neurons reached the MSCs to study the direct effect of sensory neurons on BMSCs. They found that DRG neurons enhanced osteogenic differentiation of MSCs through the activation of the Wnt/*β*-catenin signaling pathway, which was proved by the upregulation of osteogenic genes and cytoplasmic accumulation and translocation into the nucleus of *β*-catenin in BMSCs, but DRG was found to have the ability to maintain stemness of BMSCs in the coculture system in our study. It seemed different from our results. However, the BMSCs in their studies were cultured in the presence of dexamethasone, ascorbic acid, and *β*-glycerol phosphate, which could induce the osteogenic differentiation themselves without other factors [44, 45], while we did not add an osteogenic inducer in our study. Also, there was no difference in osteoblast marker genes between mono- and coculture groups without the osteogenic induction medium, which meant that the effect of the DRG neuron per se was not sufficient to induce the osteoblastogenesis in vitro. They concluded that DRG could only enhance the osteogenic differentiation in the presence of the osteogenic induction medium but could not start it. This finding was in accord with our results. In our study, the mechanism of promoting osteogenesis was that transwell coculture with DRG helped maintain the stemness of BMSCs; then, the osteogenic induction medium would show a more significant effect on osteogenic differentiation.

As was reported, in vitro culture impaired the stemness of BMSCs [46, 47] and resulted in impaired self-renewal and multilineage differentiation ability, which was not good for their use as seed cells in bone tissue engineering. Besides, the mechanism of BMSC stemness maintenance was the enhancement of their basal autophagy level in our study. This suggested that the moderate enhancement of autophagy might be beneficial to the culture of stem cells in vitro, which cleared away the impaired cellular components and kept cell homeostasis [48, 49]. Moreover, the intensity of autophagy could be regulated by certain drugs, such as rapamycin [50, 51] and bafilomycin A1 [52, 53]. Since BMSCs are widely applied as seed cells in bone tissue engineering [54], our study may provide a new strategy to expand BMSCs abundantly in vitro by regulating their autophagy intensity without impairing their self-renewal and pluripotent differentiation abilities.

It should be noted that DRG cells contained both sensory neurons and Schwann cells in our experiment, although DRG explants had been shredded and digested. These two components are similar to the sensory nerve in vivo, where sensory neurites are wrapped around by the Schwann cells [55]. Therefore, the phenomenon of stemness enhancement observed in this experiment should be considered the synthetic effects of sensory neurons and Schwann cells. In another study, it was found that Schwann cells secreted extracellular vesicles to promote and maintain the proliferation and multipotency of human dental pulp cells (hDPCs), and through proteome and Western blot analysis, they detected abundant enrichment of Oct4 and TGF*β*s in Schwann cell-derived extracellular vesicles, which explained the upregulation of stem cell-related genes and the acceleration of proliferation in hDPCs [17]. As hDPCs showed common features with BMSCs in many aspects, this result might partly explicate the stemness enhancement of BMSCs in a coculture system containing Schwann cells in our study. At the same time, it might also explain why stemness enhancement of BMSCs still existed even after blocking autophagy with compound C in the coculture group.

In our study, we used a transwell coculture system to explore the effect of DRG on BMSCs, which mimicked the implanted sensory nerve and BMSCs in TEB. And we found that DRG could help maintain the stemness of BMSCs in vitro, in which the process of secreting substance P from DRG neurons played an important role. Based on this finding, it could be hypothesized that the sensory nerves implanted into the tissue-engineered bone enhanced the autophagy of BMSCs and helped maintain their stemness, which would keep the self-renewal and multilineage differentiation abilities of BMSCs. Therefore, BMSCs would be more ready to proliferate and differentiate into other kinds of cells, such as osteoblasts, chondrocytes, vascular endothelial cells, and even Schwann cells, under the induction condition [56–58]. These cells were all critical for bone repair and regeneration. In addition, BMSCs have powerful paracrine function, including growth factors and neurotrophic factors [59–61]. And the maintenance of stemness is important for keeping this ability. Moreover, this study presented a new perspective for understanding neuronal regulation of the bone, but it still needs further study regarding which factors, except substance P, in DRG acted on BMSCs and how they worked on BMSCs.

## 5. Conclusion

DRG was helpful in maintaining the stemness of BMSCs in a transwell coculture system, and this function was achieved by enhancing the autophagy level of BSMCs through AMPK/mTOR signaling, which may explain the mechanism of the sensory nerve promoting osteogenesis, and substance P plays an important role in the process.

## Data Availability

The data used to support the findings of this study are available from the corresponding author upon request.

## Supplementary Materials

Supplementary Materials Figure S1: compound C alone did not change the autophagy level or stemness genes of BMSCs. (a) Protein expression of LC3I and LC3II. (b) Analysis of LC3II/LC3I between two groups. (c) Stem cell-related gene expression between two groups.
