# Supplementary material for: Dorsal Root Ganglion Maintains Stemness of Bone Marrow Mesenchymal Stem Cells by Enhancing Autophagy through the AMPK/mTOR Pathway in a Coculture System
Source: Stem Cells Int. 2018 Sep 30;2018:8478953. doi: 10.1155/2018/8478953 (PMC6186314; doi:10.1155/2018/8478953)
Supplement: Supplementary Materials — Figure S1: compound C alone did not change the autophagy level or stemness genes of BMSCs. (a) Protein expression of LC3I and LC3II. (b) Analysis of LC3II/LC3I between two groups. (c) Stem cell-related gene expression between two groups. [file 8478953.f1.docx]

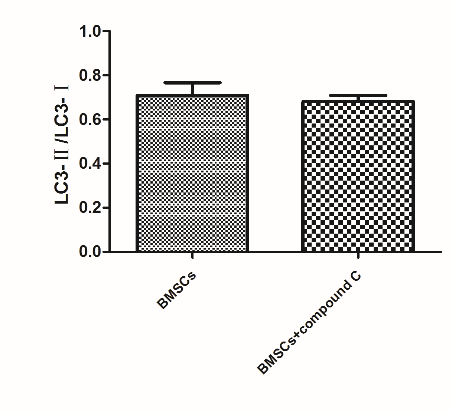

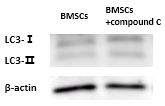

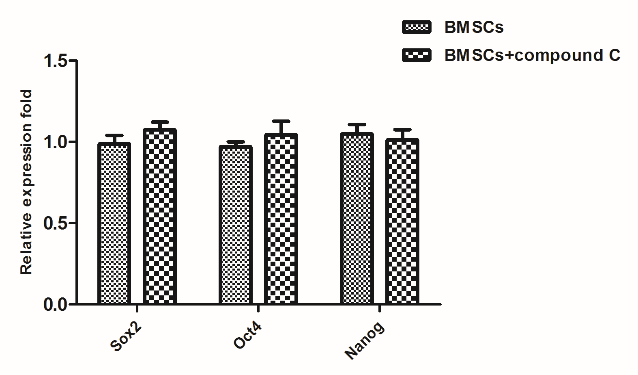


(b)

(c)

(a)

**FIGURE S1. Compound C alone didn’t change the autophagy level or stemness genes of BMSCs.**

**(a) Protein expression of LC3Ⅰand LC3Ⅱ. (b) Analysis of LC3Ⅱ/LC3Ⅰbetween two grouops.**

**(c) Stem-cells related genes expression between two groups.**
